# Evaluation of Novel Benzo-Annelated 1,4-Dihydropyridines as Potential Inhibitors of Antibacterial Efflux Pumps in *S. aureus* and MRSA Strains

**DOI:** 10.3390/ijms27093738

**Published:** 2026-04-23

**Authors:** Peter Werner, Nikoletta Szemerédi, Gabriella Spengler, Frank Erdmann, Andreas Hilgeroth

**Affiliations:** 1 Institute of Pharmacy, Martin-Luther-University Halle-Wittenberg, Wolfgang-Langenbeck-Str. 4, 06120 Halle, Germany; peter.werner@mail.de (P.W.); frank.erdmann@pharmazie.uni-halle.de (F.E.); 2 Department of Medical Microbiology, University of Szeged, Dom tér 10, 6720 Szeged, Hungary; szemeredi.nikoletta@med.u-szeged.hu (N.S.); spengler.gabriella@med.u-szeged.hu (G.S.)

**Keywords:** efflux pump inhibitor, 1,4-dihydropyridine, substituent effects, *S. aureus*, MRSA

## Abstract

Multidrug (MDR) resistances against various classes of antibiotics used in *S. aureus* and MRSA infections have emerged. With limited options for novel antibacterial compounds, there is a strong focus on finding agents against MDR phenomenon, namely causative efflux pumps. We synthesised novel benzo-annelated 1,4-dihydropyridines with various substitution patterns both at the 4- and *N*-alkyl substituents and, additionally, at the annelated aromatic residues. MDR efflux pump-inhibiting activity was evaluated in *S. aureus* strains including MRSA and was measured in a fluorescent assay system using ethidium bromide as the overall substrate of *S. aureus* efflux pumps. Favourable substituents for inhibiting efflux pump activity in *S. aureus* have been 4-methoxy and 4- and 3-chloro at the 4-phenyl position of the 1,4-dihydropyridine ring combined with an *N*-benzyl residue. The most favourable substituents for the activity inMRSA strains have been those 4-phenyl chloro substituents combined with additional pyrido residues attached to the benzo substituent at the 1,4-dihydropyridine core. Benzo-annelated 1,4-dihydropyridines are a novel class of inhibitors of MDR relevant efflux pumps in *S. aureus* strains including MRSA.

## 1. Introduction

Emerging antimicrobial resistance in bacteria means an increasing threat to global health [1,2]. Among the various pathogenic bacteria, *S. aureus* is the most prevalent one [1,3]. It causes a wide range of infections, from mild skin and soft tissue diseases to life-threatening pneumonia and endocarditis [4]. Methicillin-resistant *S. aureus* (MRSA) has become the most prominent pathogen in hospital-acquired infections [1,5,6].

Meanwhile, the increased and mostly inappropriate use of antibiotics, named as misuse in cases of unproven bacterial infection with a viral cause, has led to the resistance of *S. aureus* to multiple classes of antibiotics like aminoglycosides, macrolides and tetracyclines [7,8,9,10]. Cephalosporines have been safe antibiotics against *S. aureus* infections for a long time, but are not effective in cases of MRSA infections [3,11]. Vancomycin was a last-resort antibiotic in cases of *S. aureus* infections including MRSA. Meanwhile, increasing resistances are described [12].

So there is a strong need for novel effective antibiotics, but the list of prospective drugs is short, with two cephalosporines of the fifth generation; teicoplanin, with problems of renal toxicity, a lack of oral bioavailability, and also emerging resistances; tedizolide as a second oxazolidone antibiotic, with proven in vitro activity against MRSA; and finally tigecycline as a tetracycline with strong limitations of increasing patient mortality under application [3,13,14,15].

With the limited options of alternative novel antibiotics, there is a strong focus on combating the causes of the various antibacterial resistances. Among intrinsic *S. aureus* resistances of limiting drug uptake, modifying drug targets and enzymatically inactivating drugs, the active efflux of drugs plays a major role [1,8,16,17]. Moreover, acquired resistances both in *S. aureus* and MRSA mediated by plasmid transfer or genetic elements strengthen the phenomenon of drug resistance [18,19].

There is great diversity of active efflux pumps present in *S. aureus*. Efflux pumps from almost all families found in bacteria are described for *S. aureus* [1,19]. Early inhibitors of the efflux pump NorA have been phenothiazines, with a multifactorial activity also on altering the bacterial membrane potential [20]. Another known inhibitor of NorA is 5′-methoxyhydnocarpin. That compound is able to inhibit the efflux of berberine, with antibacterial effects towards *S. aureus* and MRSA [21,22]. The natural amide piperine was also discovered to show inhibitory activity towards the NorA efflux pump in *S. aureus*, acting synergistically with ciprofloxacin through efflux pump inhibition [23]. The natural capsaicin was demonstrated to act as a NorA efflux pump inhibitor and to reduce the invasiveness of *S. aureus* [24]. However, it is extremely difficult to find an inhibitor for all possible efflux pumps to combat efflux pump-mediated resistance. So far, no single inhibitor has reached clinical trials [1,25].

We discovered a completely novel class of benzo-annelated 1,4-dihydropyridines as inhibitors of the efflux pump activity both in *S. aureus* and MRSA depending on the various substitution patterns, as evaluated in a fluorescence assay utilising a fluorescent substrate that is transported by all of the different efflux pump families in *S. aureus* strains.

So, potent candidates have been identified with activity against *S. aureus* and MRSA. They are promising candidates for combatting the central problem of antibacterial drug resistance mediated by efflux pumps as proven inhibitors of efflux pump activity.

## 2. Results and Discussion

### 2.1. Drug Development and Synthesis

The number of efflux pump inhibitors is generally low, mostly consisting of drugs with pharmacological properties that exclude a practical use due to expected severe side effects [1,8,25]. 1,4-Ddihydropyridines of the nifedipine type have been reported to show few effects as modest inhibitors of transmembrane efflux pumps in cancer cells [26,27]. Structurally, 1,4-dihydropyridines have essential diester functions at both the 3- and the 5-position of the 1,4-dihydropyridine ring [27,28]. We wondered whether the 1,4-dihydropyridine scaffold may show effects on bacterial efflux pumps and substituted the nitrogen atom in order to minimise the suggested oxidation reactions, as described for nifedipine-type 1,4-dihydropyridines [29].

Additionally, attached phenyl residues, instead of both methyl and diester substitution, resulted in new structural compounds for evaluation as inhibitors of bacterial efflux pumps.

**Scheme 1:**
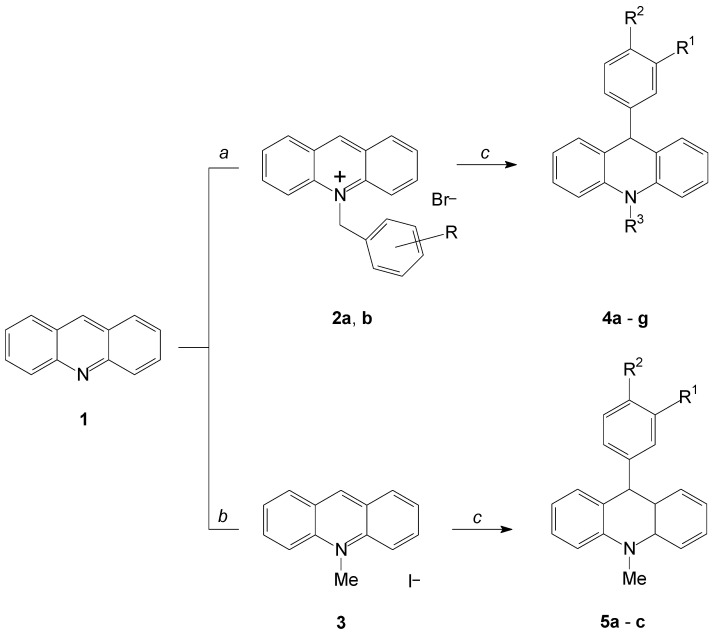
Formation of target compound series **4a**–**g** and **5a**–**c**, respectively. Reaction conditions: *a* benzyl bromide, 120 °C; *b* iodomethane, acetonitrile, reflux; *c* CuI, LiCl, Grignard reagent, room temperature.

**Scheme 2:**
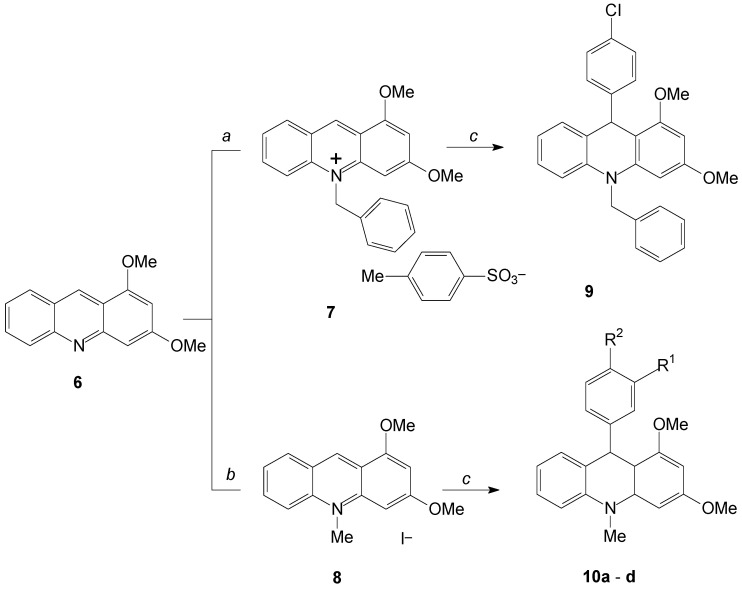
Formation of target compounds **9** and **10a**–**d**, respectively. Reaction conditions: *a* benzyltosylate, 120 °C; *b* iodomethane, sulpholane, reflux; *c* CuI, LiCl, Grignard reagent, room temperature.

The benzo-annelated 1,4-dihydropyridines **1** and **6** have been benzylated after the melting of the respective starting compound and addition of an excess of the corresponding benzyl bromide or tosylate (Scheme 1 and Scheme 2).

**Scheme 3:**
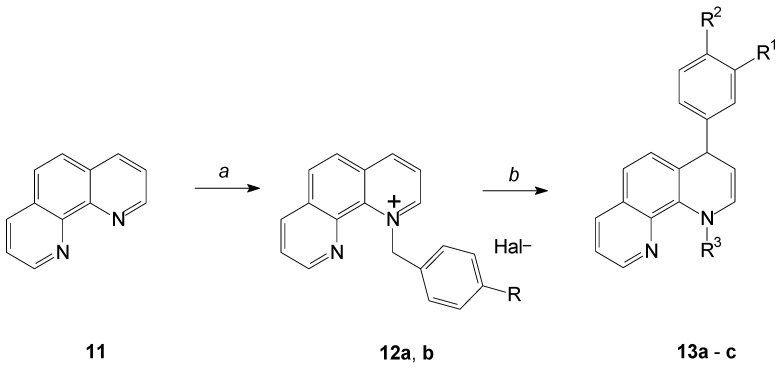
Formation of target compound series **13ac**. Reaction conditions: *a* benzyl halide, acetone, room temperature; *b* CuI, LiCl, Grignard reagent, −8 °C—room temperature.

Starting compound **11** was dissolved in acetone, and then benzyl halide was added under further stirring (Scheme 3).

For the following 4-functionalization, the resulting dried *N*-benzylated pyridinium intermediates **2a**, **b**, **7** and **12a**, **b** were dissolved in dried tetrahydrofurane under the addition of catalytic amounts of copper(I) iodide and lithium chloride. Then, an excess of the corresponding Grignard reagent had been added dropwise at room temperature to produce the final compounds **4a**–**g** and **9**, whereas compounds **13a**–**c** were yielded after Grignard reagent addition at a low temperature of −8 °C.

The methylation of the respective compounds **1** and **6** followed different conditions. While compound **1** was dissolved in acetonitrile and then treated with an excess of iodomethane for 12 h at 85 °C under reflux conditions to give the *N*-methylated compound **3**, starting compound **6** was dissolved in sulpholane and, after the addition of the excess of iodomethane, was heated under reflux conditions at 110 °C for 24 h, resulting in the respective *N*-methylated intermediate **8**. The final 4-functionalization of compounds **5a**–**c** and **10a**–**d**, respectively, followed the reaction conditions above, described for *N*-benzylated intermediate derivatives.

### 2.2. Efflux Pump Inhibition Activity of Compound Series

Compound efflux inhibition activity was determined the *S. aureus* strains including MRSA using ethidium bromide as the fluorescent substrate for almost all efflux pump types in *S. aureus* in order to identify the overall efflux pump activity of our compound series [1]. Compounds have been used in a single fixed concentration of 100 µM to determine structure-dependent effects, similar to earlier published studies on the determination of efflux pump inhibition in MRP4 in cancer cells [30]. For the calculation of each compound’s activity, the fluorescence amount was measured under inhibitor treatment. The measured amount without an inhibitor was subtracted from that amount to give the *RFI* (relative fluorescence increase) value, a mathematical difference related to the fluorescence amount without an inhibitor. The *RFI* values are shown in Table 1. In the case of efflux pump inhibition under inhibitor use, the efflux of ethidium bromide was reduced, resulting in higher fluorescence amounts within the measured bacteria and following *RFI* values > 0.

In compound class **4**, with methoxy and chloro functions attached to the phenyl residues and carboxymethyl functions attached to the *N*-benzyl residues, we determined activities towards the *S. aureus* strain that were most favourable, with dimethoxy or dichloro functions at the phenyl residues of compounds **4c** and **4g**, respectively. Compound **4g** reached an activity that was almost twofold higher than that of the used CCCP control at half of the concentration, which had to be used due to known toxic problems of cell membrane damage observed at higher concentrations. In derivative **4d**, with a carboxymethyl function at the 4-position of the *N*-benzyl residue, we found an increase in activity.

When the *N*-benzyl residue was replaced with a methyl function in compound class **5**, the dichlorophenyl compound **5c** showed the best activity. The dimethoxy functions at the molecular scaffold of derivative **9** were not favourable. Also, in compound class **10**, the dimethoxy functions at the molecular scaffold were unfavourable. All compounds **10a**–**c** generally showed poor activities, with compound **10c**’s dichlorophenyl function being the most active.

Finally, compounds **13a**–**c**, with an attached pyridyl substituent to the molecular scaffold, showed the best activities.

Compound **4c**, with dimethoxy functions at the phenyl residue, showed the best activity towards MRSA 43300 within compound class **4**. Methoxy or chloro functions at the 3-position of the phenyl residue tended to be more favourable than at the 4-position. The *N*-methyl compounds of compound class **5** were more active towards MRSA 43300 than towards the *S. aureus* strain. Again, dimethoxy functions at the molecular scaffold of compound **9** and compound class **10** had no influence on the activity compared to those of compounds **5a**–**c**. However, compounds **13a**–**c** again showed the best activities towards MRSA 43300, similar to those towards the *S. aureus* strain.

Finally, the activities towards MRSA 272123 were determined. All compounds of compound class **4** showed improved activities, with compound **4a** being the most favourable. The *N*-methyl substituent in compound class **5** was more favourable than the *N*-benzyl substituent of compound class **4**, resulting in improved activities. For compound **9**, with dimethoxy substitutions at the molecular scaffold, we found the best activity in MRSA 272123 compared to the other strains. Compounds **10a**–**d** also showed increased activities towards MRSA 272123 compared to MRSA 43300. From compound class **13**, derivative **13c** showed the best activities.

Considering the electronic nature of the varied substituents at the molecular scaffold residues, it can be stated that methoxy substituents with electron pushing effects at the phenyl residues are more effective than chloro substituents with less electron pushing effects. However, two of those substituents generally resulted in increased effects compared to one, emphasising the importance of such electronic effects.

In order to investigate the possible toxic effects of our compounds on bacteria, they were tested at 100 µM in all *S. aureus* strains without any effects on the bacterial growth, proving that they were nontoxic. Additionally, the most active compound classes, representing derivatives **4b**, **4g**, **13b** and **13c**, were tested in HEK293 mammalian kidney cells for possible cytotoxic effects in a reazurin assay to determine the degree of fluorescent resorufin formed by toxic effects of the compounds. Until a measured compound concentration of 250 µM, we observed no degree of the measured fluorescence and, consequently, no toxicity of the measured compounds.

Finally, we determined the minimum inhibitory concentration (MIC) values of the antibiotics tetracycline and ciprofloxacin in all three *S. aureus* strains and then repeated the determinations with relevant fixed concentrations of each best evaluated efflux pump inhibitor following the discussed *RFI* values (Table 2).

In the *S. aureus* strain ATCC 25923, the MIC values of tetracycline and ciprofloxacin were determined using the twofold serial dilution technique starting with a concentration of 1 µg/mL for tetracycline and 50 µg/mL for ciprofloxacin. In repeated measurements with the best inhibitors **4b**, **4g**, **13b** and **13c**, the determined MIC value for tetracycline of 0.0625 µg/mL was reduced to values <0.0019 µg/mL for all inhibitors. That means a reduction in the MIC value by a factor of 32. For ciprofloxacin, the determined MIC value of 0.78 µg/mL was reduced to 0.39 µg/mL in combination with inhibitor **13b**, with the lowest activity, whereas all the other inhibitors with higher activities resulted in determined reduced MIC values for ciprofloxacin <0.097 µg/mL. That means a reduction by a factor >8.

In MRSA strain 43300, the MIC values of tetracycline and ciprofloxacin were determined to be 0.031 µg/mL and 0.39 µg/mL, respectively. In combination with the best efflux pump inhibitors in the MRSA strain, compounds **13b** and **13c**, the MIC value for tetracycline was found to be decreased by a factor of >16 to a value <0.0019 µg/mL for compound **13b**. Compound **13c**, with a lower *RFI* value, however, was not effective, with an unchanged MIC value. The MIC value for ciprofloxacin was found decreased in combination with both compounds **13b** and **13c** to less than 0.097 µg/mL, by a factor of >4. In MRSA strain 272123, the MIC values of tetracycline and ciprofloxacin were both determined to be 25 µg/mL. Out of all the best efflux pump inhibitor compounds in that cell line, compounds **4b** and **5a** caused a decrease in the MIC values of both antibiotics to a value of 12.5 µg/mL. That means an increase in activity by a factor of 2.

Due to the proven activity of our compounds to increase ciprofloxacin and tetracycline activity, they are effective as inhibitors of ciprofloxacin-mediated transport by the MATE and NorA efflux pump and additionally of the TetA and Tet38 efflux pumps that mediate tetracycline efflux from *S. aureus* bacteria including MRSA [1]. Thus, they show a desired broad activity compared to NorA transporter-specific inhibitors like the mentioned phenothiazines, 5′-methoxyhydnocarpin, piperine or capsaicin because most antibiotics are transported by various efflux pumps [1].

## 3. Materials and Methods

General: Commercial reagents were used without further purification. The ^1^H-NMR spectra (500 MHz resp. 400 MHz) were measured using tetramethylsilane as the internal standard. Thin-layer chromatography (TLC) was performed on E. Merck 5554 silica gel plates (Merck, Darmstadt, Germany). High-resolution mass spectra were recorded using a LTQ-Orbitrap-XL mass spectrometer (Thermo Fisher Scientific, Erlangen, Germany).

**Formation of the *N*-benzylated acridinium compounds 2a**,**b** and **7**

One equivalent of acridine was melted in a flask at the respective temperature, and then one equivalent of methyl-3-(bromomethyl)-benzoate or two equivalents of methyl-4-(bromomethyl)-benzoate and two and a half equivalents of benzyltosylate, respectively, were added dropwise under stirring. The resulting solid after cooling to room temperature under stirring was washed with diethyl ether several times and then recrystallized from a mixture of methanol and diethyl ether (1:1). Finally, the product was dried over phosphorus pentoxide for 24 h.

***Data for 10-(3-(Methoxycarbonyl)benzyl)acridinium Bromide (*** **2a*)***

Yield 23%; green-yellow solid; mp 211–212 °C; ^1^H NMR (DMSO-d_6_) *δ* 10.37 (s, 1H, 9-H), 8.73 (dd, ^3^*J*_1/2,8/7_ = 8.4 Hz, ^4^*J*_1/3,8/6_ = 1.5 Hz, 2H, 1-H, 8-H), 8.62 (d, ^3^*J*_4/3,5/6_ = 9.2 Hz, 2H, 4-H, 5-H), 8.44 (ddd, ^3^*J*_3/4,6/5_ = 9.2 Hz, ^3^*J*_3/2,6/7_ = 6.8 Hz, ^4^*J*_3/1,6/8_ = 1.5 Hz, 2H, 3-H, 6-H), 8.06 (dd, ^3^*J*_2/1,7/8_ = 8.4 Hz, ^3^*J*_2/3,7/6_ = 6.8 Hz, 2H, 2-H, 7-H), 7.98 (s, 1H, 2′-H), 7.91 (d, ^3^*J*_4′/5′_ = 7.8 Hz, 1H, 4′-H), 7.43 (t, ^3^*J*_5′/4′,6′_ = 7.8 Hz, 1H, 5′-H), 7.21 (d, ^3^*J*_6′/5′_ = 7.8 Hz, 1H, 6′-H), 6.86 (s, 2H, C**H_2_**), 3.83 (s, 3H, 3′-COOC**H_3_**); MS (APCI) *m*/*z* (%) = 328.3 (100, [M-Br]^+^).

***Data for 10-(4-(Methoxycarbonyl)benzyl)acridinium Bromide (*2b*)***

Yield 29%; bright-yellow solid; mp 213–215 °C; ^1^H NMR (DMSO-d_6_) *δ* 10.37 (s, 1H, 9-H), 8.73 (dd, ^3^*J*_1/2,8/7_ = 8.4 Hz, ^4^*J*_1/3,8/6_ = 1.5 Hz, 2H, 1-H, 8-H), 8.58 (d, ^3^*J*_4/3,5/6_ = 9.2 Hz, 2H, 4-H, 5-H), 8.43 (ddd, ^3^*J*_3/4,6/5_ = 9.2 Hz, ^3^*J*_3/2,6/7_ = 6.8 Hz, ^4^*J*_3/8,6/8_ = 1.5 Hz, 2H, 3-H, 6-H), 8.09–8.02 (m, 2H, 2-H, 7-H), 7.91 (AB, *J* = 8.5 Hz, 2H, 3′-H, 5′-H), 7.30 (AB, *J* = 8.5 Hz, 2H, 2′-H, 6′-H), 6.86 (s, 2H, C**H_2_**), 3.82 (s, 3H, 4′-COOC**H_3_**); MS (APCI) *m*/*z* (%) = 328.0 (100, [M-Br]^+^).

***Data for 10-Benzyl-1,3-dimethoxyacridinium Tosylate (*7*)***

Yield 18%; red-yellow solid; mp 205–206 °C; ^1^H NMR (DMSO-d_6_) *δ* 10.05 (s, 1H, 9-H), 8.69 (dd, ^3^*J*_8/7_ = 7.8 Hz, ^4^*J*_8/6_ = 1.5 Hz, 1H, 8-H), 8.42 (d, ^3^*J*_5/6_ = 9.1 Hz, 1H, 5-H), 8.27 (ddd, ^3^*J*_6/5_ = 9.1 Hz, ^3^*J*_6/7_ = 7.8 Hz, ^4^*J*_6/8_ = 1.5 Hz, 1H, 6-H), 7.83 (t, ^3^*J*_7/6,8_ = 7.8 Hz, 1H, 7-H), 7.48 (AB, *J* = 8.1 Hz, 2H, 3″-H, 5″-H), 7.38–7.31 (m, 3H, 3′-H, 4′-H, 5′-H), 7.20 (m, 3H, 2′-H, 6′-H, 4-H), 7.10 (AB, 2H, 2″-H, 6″-H), 7.04 (AB, *J* = 1.8 Hz, 1H, 2-H), 6.52 (s, 2H, C**H_2_**), 4.14 (s, 3H, 3-OC**H_3_**), 4.10 (s, 3H, 1-OC**H_3_**), 3.17 (s, 3H, Ar-C**H_3_**); MS (APCI) *m*/*z* (%) = 330.0 (100, [M-tosylate]^+^).

***Formation of the N-methylated acridinium compounds* 3 *and* 8**

One equivalent of acridine and five equivalents of iodomethane were dissolved in acetonitrile and heated under reflux for 12 h for compound **3** or dissolved in sulpholane and heated under reflux for 24 h for compound **8**. After the removal of acetonitrile in a vacuum, the raw product **3** was washed with diethyl ether several times and recrystallized from a mixture of methanol and diethyl ether (1:1). The mixture of compound **8** was cooled down to room temperature, and then ethyl acetate was added to give a yellow precipitate that was filtered off and washed with ethyl acetate, diethyl ether, and dichloromethane. Then, it was recrystallized from a mixture of methanol and diethyl ether (1:1). Both compounds **3** and **8** were dried over phosphorus pentoxide for 24 h.

***Data for 10-Methylacridinium Iodide (*** **3*)***

Yield 50%; reddish solid; mp 219–220 °C; ^1^H NMR (DMSO-d_6_) *δ* 10.20 (s, 1H, 9-H), 8.79 (d, ^3^*J*_4/3,5/6_ = 9.3 Hz, 2H, 4-H, 5-H), 8.65 (dd, ^3^*J*_1/2,8/7_ = 7.7 Hz, ^4^*J*_1/3,8/6_ = 1.5 Hz, 2H, 1-H, 8-H), 8.47 (ddd, ^3^*J*_3/4,5/6_ = 9.3 Hz, ^3^*J*_3/2,6/7_ = 6.8 Hz, ^4^*J*_3/1,6/8_ = 1.5 Hz, 2H, 3-H, 6-H), 8.05 (dd, ^3^*J*_2/1,7/8_ = 7.7 Hz, ^3^*J*_2/3,7/6_ = 6.8 Hz, 2H, 2-H, 7-H), 4.86 (s, 3H, N-C**H_3_**); MS (APCI) *m*/*z* (%) = 194.0 (100, [M-I]^+^).

***Data for 1,3-Dimethoxy-10-methylacridinium Iodide (*** **8*)***

Yield 62%; orange solid; mp 196–197 °C; ^1^H NMR (DMSO-d_6_) *δ* 9.94 (s, 1H, 9-H), 8.63 (dd, ^3^*J*_8/7_ = 8.3 Hz, ^4^*J*_8/6_ = 1.5 Hz, 1H, 8-H), 8.59 (dd, ^3^*J*_5/6_ = 9.0 Hz, ^4^*J*_5/7_ = 0.9 Hz, 1H, 5-H), 8.32 (ddd, ^3^*J*_6/5_ = 9.0 Hz, ^3^*J*_6/7_ = 7.0 Hz, ^4^*J*_6/8_ = 1.5 Hz, 1H, 6-H), 7.90 (ddd, ^3^*J*_7/8_ = 8.3 Hz, ^3^*J*_7/6_ = 7.0 Hz, ^4^*J*_7/5_ = 0.9 Hz, 1H, 7-H), 7.33 (d, ^4^*J*_4/2_ = 1.8 Hz, 1H, 4-H), 7.02 (d, ^4^*J*_2/4_ = 1.8 Hz, 1H, 2-H), 4.62 (s, 3H, N-C**H_3_**), 4.21 (s, 3H, 1-OC**H_3_**), 4.18 (s, 3H, 3-OC**H_3_**); MS (APCI) *m*/*z* (%) = 254.0 (100, [M-I]^+^).

***Formation of the phenylated dihydroacridines* 4a**–**g*,* 5a**–**c*,* 9 *and* 10 a**–**d**

One equivalent of the respective acridinium compound was suspended in a mixture of dried THF, 0.1 equivalent of copper(I)iodide and 0.2 equivalent of lithium chloride under an argon atmosphere. Then, 3.5 equivalents of the respective Grignard reagent were added dropwise under stirring at room temperature until no more reaction product was detectable by TLC. After that, ammonium chloride solution (20%) was added and extraction with three portions of chloroform followed. The unified organic layer was extracted with ammonium chloride solution (20%), concentrated ammonia in water (1:1), hydrochloric acid solution (10%) and finally with brine. After drying over sodium sulfate and filtration, the organic layer was removed in a vacuum under reduced pressure. The remaining oil was purified through column chromatography using eluent mixtures of heptane/cyclohexane and ethyl acetate (10:1). Then, the resulting products were partly recrystallized from methanol/acetonitrile.

***Data for Methyl-3-((9-(3-methoxyphenyl)acridine-10(9H)-yl)methyl) benzoate (*** **4a*)***

Yield 31%; white solid; mp 133–135 °C; ^1^H NMR (DMSO-d_6_) *δ* 7.84 (dt, ^3^*J*_4′/5′_ = 7.7 Hz, ^4^*J*_4′/2′,6′_ = 1.5 Hz, 1H, 4′-H), 7.82 (t, ^4^*J*_2′/4′,6′_ = 1.5 Hz, 1H, 2′-H), 7.45 (t, ^3^*J*_5′/4′,6′_ = 7.7 Hz, 1H, 5′-H), 7.35 (dt, ^3^*J*_6′/5′_ = 7.7 Hz, ^4^*J*_6′/2′,4′_ = 1.5 Hz, 1H, 6′-H), 7.27 (dd, ^3^*J*_1/2,8/7_ = 7.8 Hz, ^4^*J*_1/3,8/6_ = 1.6 Hz, 2H, 1-H, 8-H), 7.14–7.11 (t, ^3^*J*_5″/4″,6″_ = 7.9 Hz, 1H, 5″-H), 7.09 (dd, ^3^*J*_3/4,6/5_ = 8.4 Hz, ^3^*J*_3/2,6/8_ = 7.4 Hz, ^4^*J*_3/1,6/8_ = 1.6 Hz, 2H, 3-H, 6-H), 6.90 (td, ^3^*J*_2/1,3;7/8,6_ = 7.4 Hz, ^4^*J*_2/4,7/5_ = 1.0 Hz, 2H, 2-H, 7-H), 6.80 (dd, ^3^*J*_4/3,5/6_ = 8.4 Hz, ^4^*J*_4/2,5/7_ = 1.0 Hz, 2H, 4-H, 5-H), 6.69 (m, 2H, 4″-H, 6″-H), 6.67 (m, 1H, 2″-H), 5.36 (s, 1H, 9-H), 5.32 (s, 2H, C**H_2_**), 3.80 (s, 3H, 3′-COOC**H_3_**), 3.65 (s, 3H, 3″-OC**H_3_**); HRMS (ESI) *m*/*z* (%) = 436.190 (100, [M + H]^+^), calculated for [C_29_H_26_NO_3_]^+^: 436.191.

***Data for Methyl-3-((9-(4-methoxyphenyl)acridine-10(9H)-yl)methyl) benzoate (*** **4b*)***

Yield 35%; white solid; mp 158–160 °C; ^1^H NMR (DMSO-d_6_) *δ* 7.84 (dt, ^3^*J*_4′/5′_ = 7.8 Hz, ^4^*J*_4′/2′,6′_ = 1.9 Hz, 1H, 4′-H), 7.82 (t, ^4^*J*_2′/4′,6′_ = 1.9 Hz, 1H, 2′-H), 7.45 (t, ^3^*J*_5′/4′,6′_ = 7.8 Hz, 1H, 5′-H), 7.35 (dt, ^3^*J*_6′/5′_ = 7.8 Hz, ^4^*J*_6′/2′,4′_ = 1.9 Hz, 1H, 6′-H), 7.24 (dd, ^3^*J*_1/2,8/7_ = 7.4 Hz, ^4^*J*_1/3, 8/6_ = 1.6 Hz, 2H, 1-H, 8-H), 7.08 (ddd, ^3^*J*_3/4,6/5_ = 8.4 Hz, ^3^*J*_3/2,6/7_ = 7.4 Hz, ^4^*J*_3/1,6/8_ = 1.6 Hz, 2H, 3-H, 6-H), 7.01 (AB, *J* = 8.6 Hz, 2H, 2″-H, 6″-H), 6.89 (td, ^3^*J*_2/1,3;7/8,6_ = 7.4 Hz, ^4^*J*_2/4,7/5_ = 1.1 Hz, 2H, 2-H, 7-H), 6.80 (d, ^3^*J*_4/3,5/6_ = 8.4 Hz, ^4^*J*_4/2,5/7_ = 1.1 Hz, 2H, 4-H, 5-H), 6.76 (AB, *J* = 8.6 Hz, 2H, 3″-H, 5″-H), 5.33 (s, 1H, 9-H), 5.31 (s, 2H, C**H_2_**), 3.80 (s, 3H, 3′-COOC**H_3_**), 3.66 (s, 3H, 4″-OC**H_3_**); HRMS (ESI) *m*/*z* (%) = 436.1906 (100, [M + H]^+^), calculated for [C_29_H_26_NO_3_]^+^: 436.1907.

***Data for Methyl-3-((9-(3,4-dimethoxyphenyl)acridine-10(9H)-yl)methyl) benzoate (*** **4c*)***

Yield 43%; white solid; mp 135–136 °C; ^1^H NMR (DMSO-d_6_) *δ* 7.84 (dd, ^3^*J*_4′/5′_ = 7.8 Hz, ^4^*J*_4′/2′,6′_ = 2.0 Hz, 1H, 4′-H), 7.82 (t, ^4^*J*_2′/4′/6′_ = 2.0 Hz, 1H, 2′-H), 7.46 (t, ^3^*J*_5′/4′,6′_ = 7.8 Hz, 1H, 5′-H), 7.37 (dt, ^3^*J*_6′/5′_ = 7.8 Hz, ^4^*J*_6′/2′/4′_ = 2.0 Hz, 1H, 6′-H), 7.27 (dd, ^3^*J*_1/2,8/7_ = 7.7 Hz, ^4^*J*_1/3,8/6_ = 1.6 Hz, 2H, 1-H, 8-H), 7.09 (ddd, ^3^*J*_3/4,6/5_ = 8.5 Hz, ^3^*J*_3/2,6/7_ = 7.3 Hz, ^4^*J*_3/1,6/8_ = 1.6 Hz, 2H, 3-H, 6-H), 6.89 (td, ^3^*J*_2/1,3;7/8,6_ = 7.3 Hz, ^4^*J*_2/4,7/5_ = 1.0 Hz, 2H, 2-H, 7-H), 6.82–6.78 (m, 3H, 2″-H, 4-H, 5-H), 6.76 (d, ^3^*J*_5″/6″_ = 8.4 Hz, 1H, 5″-H), 6.53 (dd, ^3^*J*_6″/5″_ = 8.4 Hz, ^4^*J*_6″/2″_ = 2.0 Hz, 1H, 6″-H), 5.32 (s, 2H, C**H_2_**), 5.31 (s, 1H, 9-H), 3.80 (s, 3H, 3′-COOC**H_3_**), 3.65 (s, 3H, 3″-OC**H_3_**), 3.63 (s, 3H, 4″-OC**H_3_**); HRMS (ESI) *m*/*z* (%) = 488.182 (100, [M + Na]^+^), calculated for [C_30_H_27_NO_4_Na]^+^: 488.184.

***Data for Methyl-4-((9-(3,4-dimethoxyphenyl)acridine-10(9H)-yl)methyl benzoate (*** **4d*)***

Yield 29%; white solid; mp 160–161 °C; ^1^H NMR (DMSO-d_6_) *δ* 7.90 (AB, *J* = 8.4 Hz, 2H, 3′-H, 5′-H), 7.30–7.23 (m, 4H, 1-H, 8-H, 2′-H, 6′-H), 7.09 (ddd, ^3^*J*_3/4,6/5_ = 8.5 Hz, ^3^*J*_3/2,6/7_ = 7.4 Hz, ^4^*J*_3/1,6/8_ = 1.6 Hz, 2H, 3-H, 6-H), 6.90 (td, ^3^*J*_2/1,3;7/8,6_ = 7.4 Hz, ^4^*J*_2/4,7/5_ = 1.1 Hz, 2H, 2-H, 7-H), 6.81–6.74 (m, 4H, 4-H, 5-H, 2″-H, 5″-H), 6.54 (dd, ^3^*J*_6″/5″_ = 8.3 Hz, ^4^*J*_6″/2″_ = 2.1 Hz, 1H, 6″-H), 5.32 (s, 2H, C**H_2_**), 5.30 (s, 1H, 9-H), 3.83 (s, 3H, 4′-COOC**H_3_**), 3.66 (s, 3H, 4″-OC**H_3_**), 3.63 (s, 3H, 3″-OC**H_3_**); HRMS (ESI) *m*/*z* (%) = 464.1852 (100, [M-H]^+^), calculated for [C_30_H_26_NO_4_]^+^: 464.1862.

***Data for Methyl-3-((9-(3-chlorophenyl)acridine-10(9H)-yl)methyl )benzoate (*** **4e*)***

Yield 33%; white solid; mp 137–138 °C; ^1^H NMR (DMSO-d_6_) *δ* 7.84 (dt, ^3^*J*_4′/5′_ = 7.8 Hz, ^4^*J*_4′/2′,6′_ = 2.0 Hz, 1H, 4′-H), 7.81 (t, ^4^*J*_2′/4′,6′_ = 2.0 Hz, 1H, 2′-H), 7.45 (t, ^3^*J*_5′/6′_ = 7.8 Hz, 1H, 5′-H), 7.34 (dd, ^3^*J*_6′/5′_ = 7.8 Hz, ^4^*J*_6′/2′,4′_ = 2.0 Hz, 1H, 6′-H), 7.31 (dd, ^3^*J*_1/2,8/7_ = 7.4 Hz, ^4^*J*_1/3,8/6_ = 1.6 Hz, 2H, 1-H, 8-H), 7.24 (t, ^3^*J*_5″/4″,6″_ = 7.7 Hz, 1H, 5″-H), 7.21–7.17 (m, 1H, 4″-H), 7.16–7.10 (m, 3H, 2″-H, 3-H, 6-H), 7.06 (dt, ^3^*J*_6″/5″_ = 7.7 Hz, ^4^*J*_6″/2″,4″_ = 1.5 Hz, 1H, 6″-H), 6.93 (td, ^3^*J*_2/1,3;7/8,6_ = 7.4 Hz, ^4^*J*_2/4, 6/5_ = 1.0 Hz, 2H, 2-H, 7-H), 6.88–6.82 (dd, ^3^*J*_4/3, 5/6_ = 8.3 Hz, ^4^*J*_4/2, 5/7_ = 1.0 Hz, 2H, 4-H, 5-H), 5.47 (s, 1H, 9-H), 5.34 (s, 2H, **CH_2_**), 3.80 (s, 3H, 3′-COO**CH_3_**); HRMS (ESI) *m*/*z* (%) = 438.1254 (100, [M-H]^+^), calculated for [C_28_H_21_ClNO_2_]^+^: 438.1255.

***Data for Methyl-3-((9-(4-chlorophenyl)acridine-10(9H)-yl)methyl) benzoate (*** **4f*)***

Yield 32%; white solid; mp 149–151 °C; ^1^H NMR (DMSO-d_6_) *δ* 7.84 (dt, ^3^*J*_4′/5′_ = 7.8 Hz, ^4^*J*_4′/2′,6′_ = 2.0 Hz, 1H, 4′-H), 7.80 (t, ^4^*J*_2′/4′,6′_ = 2.0 Hz, 1H, 2′-H), 7.45 (t, ^3^*J*_5′/4′,6′_ = 7.8 Hz, 1H, 5′-H), 7.34 (dt, ^3^*J*_6′/5′_ = 7.8 Hz, ^4^*J*_6′/2′,4′_ = 2.0 Hz, 1H, 6′-H), 7.30–7.23 (m, 4H, 1-H, 8-H, 2″-H, 6″-H), 7.15–7.08 (m, 4H, 3-H, 6-H, 3″-H, 5″-H), 6.91 (td, ^3^*J*_2/1,3;7/8,6_ = 7.4 Hz, ^4^*J*_2/4,7/4_ = 1.0 Hz, 2H, 2-H, 7-H), 6.83 (dd, ^3^*J*_4/3,5/6_ = 8.3 Hz, ^4^*J*_4/2,5/7_ = 1.0 Hz, 2H, 4-H, 5-H), 5.44 (s, 1H, 9-H), 5.32 (s, 2H, C**H_2_**), 3.80 (s, 3H, 3′-COOC**H_3_**); HRMS (ESI) *m*/*z* (%) = 462.1231 (100, [M + Na]^+^), calculated for [C_28_H_22_ClNO_2_Na]^+^: 462.1237.

***Data for Methyl-3-((9-(3,4-dichlorophenyl)acridine-10(9H)-yl)methyl) benzoate (*** **4g*)***

Yield 65%; white solid; mp 157–158 °C; ^1^H NMR (DMSO-d_6_) *δ* 7.86–7.81 (dd, ^3^*J*_4′/5′_ = 7.8 Hz, ^4^*J*_4′/2′_ = 2.8 Hz, 1H, 4′-H), 7.79 (t, ^4^*J*_2′/4′,6′_ = 2.8 Hz, 1H, 2′-H), 7.47 (d, ^3^*J*_5″/6″_ = 8.4 Hz, 1H, 5″-H), 7.45 (t, ^3^*J*_5′/4′,6′_ = 7.8 Hz, 1H, 5′-H), 7.38–7.29 (m, 4H, 1-H, 8-H, 6′-H, 2″-H), 7.14 (dd, ^3^*J*_3/4, 6/5_ = 8.4 Hz, ^3^*J*_3/2, 6/7_ = 7.4 Hz, 2H, 3-H, 6-H), 7.05 (dd, ^3^*J*_6″/5″_ = 8.4 Hz, ^4^*J*_6″/2″_ = 2.2 Hz, 1H, 6″-H), 6.93 (t, ^3^*J*_2/1,3; 7/8,6_ = 7.4 Hz, 2H, 2-H, 7-H), 6.86 (d, ^3^*J*_4/3, 5/6_ = 8.4 Hz, 2H, 4-H, 5-H), 5.50 (s, 1H, 9-H), 5.33 (s, 2H, C**H_2_**), 3.80 (s, 3H, COOC**H_3_**); HRMS (ESI) *m*/*z* (%) = 472.0862 (100, [M-H]^+^), calculated for [C_28_H_20_Cl_2_NO_2_]^+^: 472.0866.

***Data for 9-(3-Chlorophenyl)-10-methyl-9,10-dihydroacridine (*** **5a*)***

Yield 46%; white solid; mp 105–106 °C; ^1^H NMR (DMSO-d_6_) *δ* 7.31 (dd, ^3^*J*_1/2,8/7_ = 7.4 Hz, ^4^*J*_1/3,8/6_ = 1.7 Hz, 2H, 1-H, 8-H), 7.26 (ddd, ^3^*J*_3/4,6/5_ = 8.2 Hz, ^3^*J*_3/2,6/7_ = 7.4 Hz, ^4^*J*_3/1,6/8_ = 1.7 Hz, 2H, 3-H, 6-H), 7.21 (t, ^3^*J*_5′/4′,6′_ = 7.8 Hz, 1H, 5′-H), 7.16 (ddd, *J* = 7.8 Hz, ^4^*J*_4′/6′_ = 2.2 Hz, ^4^*J*_4′/2′_ = 1.2 Hz, 1H, 4′-H), 7.10–7.07 (m, 3H, 2′-H, 4-H, 5-H), 7.02–6.99 (m, 1H, 6′-H), 6.95 (td, ^3^*J*_2/1,3;7/8,6_ = 7.4 Hz, ^4^*J*_2/4,7/5_ = 1.1 Hz, 2H, 2-H, 7-H), 5.34 (s, 1H, 9-H), 3.37 (s, 3H, N-C**H_3_**); HRMS (ESI) *m*/*z* (%) = 304.0888 (100, [M-H]^+^), calculated for [C_20_H_15_ClN]^+^: 304.0888.

***Data for 9-(4-Chlorophenyl)-10-methyl-9,10-dihydroacridine (*** **5b*)***

Yield 53%; white solid; mp 129–131 °C; ^1^H NMR (DMSO-d_6_) *δ* 7.31–7.25 (m, 4H, 1-H, 3-H, 6-H, 8-H), 7.23 (AB, *J* = 8.4 Hz, 2H, 3′-H, 5′-H), 7.07 (dd, ^3^*J*_4/3,5/6_ = 8.1 Hz, ^3^*J*_4/2,5/7_ = 1.1 Hz, 2H, 4-H, 5-H), 7.07 (AB, *J* = 8.4 Hz, 2H, 2′-H, 6′-H) 6.94 (td, ^3^*J*_2/1,3;7/8,6_ = 7.4 Hz, ^4^*J*_2/4,7/5_ = 1.1 Hz, 2H, 2-H, 7-H), 5.31 (s, 1H, 9-H), 3.36 (s, 3H, N-C**H_3_**); HRMS (ESI) *m*/*z* (%) = 304.0888 (100, [M-H]^+^), calculated for [C_20_H_15_ClN]^+^: 304.0888.

***Data for 9-(3,4-Dichlorophenyl)-10-methyl-9,10-dihydroacridine (*** **5c*)***

Yield 41%; white solid; mp 129–131 °C; ^1^H NMR (DMSO-d_6_) *δ* 7.44 (d, ^3^*J*_5′/6′_ = 8.5 Hz, 1H, 5′-H), 7.32 (dd, ^3^*J*_1/2,8/7_ = 7.5 Hz, ^4^*J*_1/3,8/6_ = 1.8 Hz, 2H, 1-H, 8-H), 7.30–7.23 (m, 3H, 2′-H, 3-H, 6-H), 7.10 (d, ^3^*J*_4/3,5/6_ = 8.3 Hz, 2H, 4-H, 5-H), 7.02–6.93 (m, 3H, 2-H, 7-H, 6′-H), 5.37 (s, 1H, 9-H), 3.37 (s, 3H, N-C**H_3_**); HRMS (ESI) *m*/*z* (%) = 338.0493 (100, [M-H]^+^), calculated for [C_20_H_14_Cl_2_N]^+^: 338.0498.

***Data for 10-Benzyl-9-(4-chlorophenyl)-1,3-dimethoxy-9,10-dihydroacridine (*** **9*)***

Yield 61%; white solid; mp 129–131 °C; ^1^H NMR (DMSO-d_6_) *δ* 7.36 (dd, ^3^*J*_8/7_ = 7.5 Hz, ^4^*J*_8/6_ = 1.6 Hz, 1H, 8-H), 7.30–7.26 (AB, 2H, 3″-H, 5″-H), 7.25–7.19 (m, 3H, 3′-H, 4′-H, 5′-H), 7.12–7.08 (m, 4H, 2′-H, 2″-H, 6′-H, 6″-H), 7.08–7.04 (m, 1H, 5-H), 6.87 (ddd, ^3^*J*_6/5_ = 8.1 Hz, ^3^*J*_6/7_ = 7.5 Hz, ^4^*J*_6/8_ = 1.6 Hz, 1H, 6-H), 6.80 (d, ^3^*J*_7/6,8_ = 7.5 Hz, 1H, 7-H), 6.23 (d, ^4^*J*_4/2_ = 2.2 Hz, 1H, 4-H), 6.04 (d, ^4^*J*_2/4_ = 2.2 Hz, 1H, 2-H), 5.48 (s, 1H, 9-H), 5.20 (s, 2H, C**H_2_**), 3.76 (s, 3H, 3-OC**H_3_**), 3.61 (s, 3H, 1-OC**H_3_**); HRMS (ESI) *m*/*z* (%) = 442.1568 (100, [M + H]^+^), calculated for [C_28_H_25_ClNO_2_]^+^: 442.1574.

***Data for 9-(3-Chlorophenyl)-1,3-dimethoxy-10-methyl-9,10-dihydroacridine (*** **10a*)***

Yield 57%; white solid; mp 145–147 °C; ^1^H NMR (DMSO-d_6_) *δ* 7.40 (dd, ^3^*J*_8/7_ = 7.4 Hz, ^4^*J*_8/6_ = 1.6 Hz, 1H, 8-H), 7.22 (td, ^3^*J*_6/7_ = 7.5 Hz, ^4^*J*_6/8_ = 1.6 Hz, 1H, 6-H), 7.18 (t, ^3^*J*_5′/6′,4′_ = 8.0 Hz, 1H, 5′-H), 7.12 (ddd, ^3^*J*_4′/5′_ = 8.0 Hz, ^4^*J*_4′/2′,6′_ = 2.0 Hz, 1H, 4′-H), 7.05 (t, ^4^*J*_2′/4′,6′_ = 2.0 Hz, 1H, 2′-H), 7.05–7.02 (m, 1H, 5-H), 7.02–6.99 (m, 1H, 6′-H), 6.93 (td, ^3^*J*_7/6,8_ = 7.5 Hz, ^4^*J*_7/5_ = 1.1 Hz, 1H, 7-H), 6.29 (d, ^4^*J*_4/2_ = 2.2 Hz, 1H, 4-H), 6.27 (d, ^4^*J*_2/4_ = 2.2 Hz, 1H, 2-H) 5.46 (s, 1H, 9-H), 3.81 (s, 3H, 3-OC**H_3_**), 3.77 (s, 3H, 1-OC**H_3_**), 3.35 (s, 3H, N-C**H_3_**); HRMS (ESI) *m*/*z* (%) = 364.1097 (100, [M-H]^+^), calculated for [C_22_H_19_ClNO_2_]^+^: 364.1099.

***Data for 9-(4-Chlorophenyl)-1,3-dimethoxy-10-methyl-9,10-dihydroacridine (*** **10b*)***

Yield 26%; white solid; mp 149–150 °C; ^1^H NMR (DMSO-d_6_) *δ* 7.36 (dd, ^3^*J*_8/7_ = 7.5 Hz, ^4^*J*_8/6_ = 1.6 Hz, 1H, 8-H), 7.22–7.17 (m, 3H, 6-H, 2′-H, 6′-H), 7.05 (AB, *J* = 8.7 Hz, 2H, 3′-H, 5′-H), 7.02 (dd, ^3^*J*_5/6_ = 8.4 Hz, ^4^*J*_5/7_ = 1.1 Hz. 1H, 5-H), 6.92 (td, ^3^*J*_7/6,8_ = 7.5 Hz, ^4^*J*_7/5_ = 1.1 Hz, 1H, 7-H), 6.28 (d, ^4^*J*_4/2_ = 2.2 Hz, 1H, 4-H), 6.26 (d, ^4^*J*_2/4_ = 2.2 Hz, 1H, 2-H), 5.43 (s, 1H, 9-H), 3.80 (s, 3H, 3-OC**H_3_**), 3.76 (s, 3H, 1-OC**H_3_**), 3.34 (s, 3H, N-C**H_3_**); HRMS (ESI) *m*/*z* (%) = 366.1252 (100, [M + H]^+^), calculated for [C_22_H_21_ClNO_2_]^+^: 366.1255.

***Data for 9-(3,4-Dichlorophenyl)-1,3-dimethoxy-10-methyl-1,9-dihydroacridine (*** **10c*)***

Yield 26%; white solid; mp 110–112 °C; ^1^H NMR (DMSO-d_6_) *δ* 7.42 (dd, ^3^*J*_8/7_ = 7.5 Hz, ^4^*J*_8/6_ = 1.5 Hz, 1H, 8-H), 7.41 (d, ^3^*J*_5′/6′_ = 8.5 Hz, 1H, 5′-H), 7.24 (d, ^4^*J*_2′/6′_ = 2.0 Hz, 1H, 2′-H), 7.22 (ddd, ^3^*J*_6/5_ = 8.5 Hz, ^3^*J*_6/7_ = 7.5 Hz, ^4^*J*_6/8_ = 1.5 Hz, 1H, 6-H), 7.04 (dd, ^3^*J*_5/6_ = 8.5 Hz, ^4^*J*_5/7_ = 1.1 Hz, 1H, 5-H), 7.00 (dd, ^3^*J*_6′/5′_ = 8.5 Hz, ^4^*J*_6′/2′_ = 2.1 Hz, 1H, 6′-H), 6.93 (td, ^3^*J*_7/6,8_ = 7.5 Hz, ^4^*J*_7/5_ = 1.1 Hz, 1H, 7-H), 6.29 (AB, *J* = 2.1 Hz, 1H, 4-H), 6.28 (AB, *J* = 2.1 Hz, 1H, 2-H), 5.47 (s, 1H, 9-H), 3.81 (s, 3H, 1-OC**H_3_**), 3.78 (s, 3H, 3-OC**H_3_**), 3.35 (s, 3H, N-C**H_3_**); HRMS (ESI) *m*/*z* (%) = 400.0864 (100, [M + H]^+^), calculated for [C_22_H_20_Cl_2_NO_2_]^+^: 400.0866.

***Data for 9-(3,4-Dimethoxyphenyl)-1,3-dimethoxy-10-methyl-1,9-dihydroacridine (*** **10d*)***

Yield 32%; white solid; mp 144–145 °C; ^1^H NMR (DMSO-d_6_) *δ* 7.34 (dd, ^3^*J*_8/7_ = 7.4 Hz, ^4^*J*_8/6_ = 1.6 Hz, 1H, 8-H), 7.19 (ddd, ^3^*J*_6/5_ = 8.1 Hz, ^3^*J*_6/7_ = 7.4 Hz, ^4^*J*_6/8_ = 1.6 Hz, 1H, 6-H), 7.00 (dd, ^3^*J*_5/6_ = 8.1 Hz, ^4^*J*_5/7_ = 1.1 Hz, 1H, 5-H), 6.90 (td, ^3^*J*_7/8,6_ = 7.4 Hz, ^4^*J*_7/5_ = 1.1 Hz, 1H, 7-H), 6.77 (d, ^4^*J*_2′/6′_ = 2.1 Hz, 1H, 2′-H), 6.68 (d, ^3^*J*_5′/6′_ = 8.3 Hz, 1H, 5′-H), 6.43 (dd, ^3^*J*_6′/5′_ = 8.3 Hz, ^4^*J*_6′/2′_ = 2.1 Hz, 1H, 6′-H), 6.26 (AB, *J* = 2.2 Hz, 1H, 4-H), 6.25 (AB, *J* = 2.2 Hz, 1H, 2-H), 5.35 (s, 1H, 9-H), 3.79 (s, 3H, 1-OC**H_3_**), 3.78 (s, 3H, 3-OC**H_3_**), 3.61 (s, 3H, 4′-OC**H_3_**), 3.60 (s, 3H, 3′-OC**H_3_**), 3.35 (s, 3H, N-C**H_3_**); HRMS (ESI) *m*/*z* (%) = 392.1854 (100, [M + H]^+^), calculated for [C_24_H_26_NO_4_]^+^: 392.1856.

***Formation of the N-benzylated phenanthrolinium compounds* 12a *and* b**

One equivalent of phenantroline was dissolved in acetone, one equivalent of benzyl halogenide was added, and the mixture stirred at room temperature for 24 h. Then, the solvent was removed, and the remaining precipitate was washed with diethyl ether and recrystallized from a mixture of methanol and diethyl ether (1:1). Both compounds were dried over phosphorus pentoxide for 24 h.

***Data for 1-Benzyl-1,10-phenanthrolium bromide (*** **12a*)***

Yield 74%; white yellow solid; mp 144–145 °C; ^1^H NMR (DMSO-d_6_) *δ* 9.77 (dd, ^3^*J*_2/3_ = 5.9 Hz, ^4^*J*_2/4_ = 1.5 Hz, 1H, 2-H), 9.52 (dd, ^3^*J*_4/3_ = 8.2 Hz, ^4^*J*_4/2_ = 1.5 Hz, 1H, 4-H), 9.21 (dd, ^3^*J*_9/8_ = 4.3 Hz, ^4^*J*_9/7_ = 1.8 Hz, 1H, 9-H), 8.76 (dd, ^3^*J*_7/8_ = 8.2 Hz, ^4^*J*_7/9_ = 1.8 Hz, 1H, 7-H), 8.54 (dd, ^3^*J*_3/4_ = 8.2 Hz, ^3^*J*_3/2_ = 5.9 Hz, 1H, 3-H), 8.45 (s, 2H, 5-H, 6-H), 8.01 (dd, ^3^*J*_8/7_ = 8.2 Hz, ^3^*J*_8/9_ = 4.3 Hz, 1H, 8-H), 7.34 (s, 2H, C**H_2_**), 7.32–7.22 (m, 5H, 2′-H, 3′-H, 4′-H, 5′-H, 6′-H); MS (APCI) *m*/*z* (%) = 271.3 (100, [M-Br]^+^).

***Data for 1-(4-Methoxybenzyl)-1,10-phenanthrolinium iodide (*** **12b*)***

Yield 98%; green solid; mp 128–129 °C; ^1^H NMR (DMSO-d_6_) *δ* 9.73 (dd, ^3^*J*_2/3_ = 6.0 Hz, ^4^*J*_2/4_ = 1.5 Hz, 1H, 2-H), 9.47 (dd, ^3^*J*_4/3_ = 8.2 Hz, ^4^*J*_4/2_ = 1.5 Hz, 1H, 4-H), 9.11 (dd, ^3^*J*_9/8_ = 4.3 Hz, ^4^*J*_9/7_ = 1.8 Hz, 1H, 9-H), 8.77 (dd, ^3^*J*_7/8_ = 8.2 Hz, ^4^*J*_7/9_ = 1.8 Hz, 1H, 7-H), 8.51–8.50 (m, 1H, 3-H), 8.43 (d, ^3^*J*_5/6_ = 2.2 Hz, 2H, 5-H, 6-H), 8.04 (dd, ^3^*J*_8/7_ = 8.2 Hz, ^3^*J*_8/9_ = 4.3 Hz, 1H, 8-H), 7.29 (s, 2H, C**H_2_**), 7.28–7.24 (AB, *J* = 8.8 Hz, 2H, 2′-H, 6′-H), 6.87–6.82 (AB, *J* = 8.8 Hz, 2H, 3′-H, 5′-H); MS (APCI) *m*/*z* (%) = 301.1 (100, [M-I]^+^).

***Formation of the phenylated dihydrophenanthrolines* 13 a**–**c**

One equivalent of the respective phenanthrolinium compound was suspended in a mixture of dried THF, 0.1 equivalent of copper(I)iodide and 0.2 equivalent of lithium chloride under an argon atmosphere. The mixture was cooled down to −8 °C and stirred for 5 min at that temperature. Then, four equivalents of the respective Grignard reagent were added dropwise under stirring at −8 °C for 15 min and additionally for 30 min at room temperature. The work-up procedure followed, as described above for the phenylated dihydroacridines. The final remaining oil was purified through column chromatography using eluent mixtures of heptane and chloroform (1:1) for compound **13a** and heptane, toluene and ethyl acetate (10:1:1) for **13b** and **c**. Then, the resulting oily products were recrystallized from diethylether/methanol (**13a**) and acetone/water at −18 °C (**13b** and **c**).

***Data for 1-Benzyl-4-(4-chlorophenyl)-1,4-dihydro-1,10-phenanthroline (*** **13a*)***

Yield 25%; pale-yellow solid; mp 80–82 °C; ^1^H NMR (DMSO-d_6_) *δ* 8.84 (dd, ^3^*J*_9/8_ = 4.1 Hz, ^4^*J*_9/7_ = 1.8 Hz, 1H, 9-H), 8.19 (dd, ^3^*J*_7/8_ = 8.3 Hz, ^4^*J*_7/9_ = 1.8 Hz, 1H, 7-H), 7.46–7.41 (m, 3H, 8-H, 3″-H, 5″-H), 7.37–7.31 (m, 3H, 6-H, 2′-H, 6′-H), 7.29–7.22 (m, 5H, 5-H, 3′-H, 5′-H, 2″-H, 6″-H), 7.22–7.15 (m, 1H, 4′-H), 6.71 (d, ^3^*J*_2/3_ = 9.3 Hz, 1H, 2-H), 6.17 (dd, ^3^*J*_3/2_ = 9.3 Hz, ^3^*J*_3/4_ = 6.2 Hz, 1H, 3-H), 5.22 (AB, *J_A_*_/*B*_ = 15.2 Hz, 2H, C**H_2_**), 5.11 (d, ^3^*J*_4/3_ = 6.2 Hz, 1H, 4-H); HRMS (ESI) *m*/*z* (%) = 381.1153 (100, [M-H]^+^), calculated for [C_25_H_18_ClN_2_]^+^: 381.1153.

***Data for 1-(4-Methoxybenzyl)-4-(4-chlorophenyl)-1,4-dihydro-1,10-phenanthroline (*** **13b*)***

Yield 25%; bright-yellow solid; mp 93–94 °C; ^1^H NMR (DMSO-d_6_) *δ* 8.86 (dd, ^3^*J*_9/8_ = 4.1 Hz, ^4^*J*_9/7_ = 1.8 Hz, 1H, 9-H), 8.19 (dd, ^3^*J*_7/8_ = 8.2 Hz, ^4^*J*_7/9_ = 1.8 Hz, 1H, 7-H), 7.44 (dd, ^3^*J*_8/7_ = 8.2 Hz, ^3^*J*_8/9_ = 4.1 Hz, 1H, 8-H), 7.37 (m, 2H, 3″-H, 5″-H), 7.33 (m, 3H, 2′-H, 6′-H, 6-H), 7.27 (d, ^3^*J*_5/6_ = 8.2 Hz, 1H, 5-H), 7.23 (m, 2H, 2″-H, 6″-H), 6.81 (m, 2H, 3′-H, 5′-H), 6.70 (d, ^3^*J*_2/3_ = 9.2 Hz, 1H, 2-H), 6.17 (dd, ^3^*J*_3/2_ = 9.2 Hz, ^3^*J*_3/4_ = 6.2 Hz, 1H, 3-H), 5.10 (AB, *J* = 14.8 Hz, 1H, C**H_2_**), 5.09 (d, ^3^*J*_4/3_ = 6.2 Hz, 1H, 4-H), 3.69 (s, 3H, OC**H_3_**); HRMS (ESI) *m*/*z* (%) = 413.1414 (100, [M + H]^+^), calculated for [C_26_H_22_ClN_2_O]^+^: 413.1415.

***Data for 1-(4-Methoxybenzyl)-4-(3,4-dichlorophenyl)-1,4-dihydro-1,10-phenan- throline (*** **13c*)***

Yield 41%; bright-yellow solid; mp 72–73 °C; ^1^H NMR (DMSO-d_6_) *δ* 8.89 (dd, ^3^*J*_9/8_ = 4.2 Hz, ^4^*J*_9/7_ = 1.9 Hz, 1H, 9-H), 8.22 (dd, ^3^*J*_7/8_ = 8.3 Hz, ^4^*J*_7/9_ = 1.8 Hz, 1H, 7-H), 7.52–7.50 (m, 1H, 6″-H), 7.48–7.45 (m, 1H, 8-H), 7.45–7.42 (m, 1H, 5″-H), 7.40–7.36 (m, 3H, 2′-H, 6-H, 6′-H), 7.32–7.26 (m, 2H, 2″-H, 5-H), 6.82 (AB, *J* = 8.5 Hz, 2H, 3′-H, 5′-H), 6.73 (d, ^3^*J*_2/3_ = 9.2 Hz, 1H, 2-H), 6.21 (dd, ^3^*J*_3/2_ = 9.2 Hz, ^3^*J*_3/4_ = 6.3 Hz, 1H, 3-H), 5.10 (d, *J* = 14.7 Hz, 1H, C**H_2_**), 5.12 (d, ^3^*J*_4/3_ = 6.3 Hz, 1H, 4-H), 3.69 (s, 3H, OC**H_3_**); HRMS (ESI) *m*/*z* (%) = 447.1021 (100, [M + H]^+^), calculated for [C_26_H_21_Cl_2_N_2_O]^+^: 447.1025.

**Efflux Pump Inhibition Assay**

The ability of the tested compounds to inhibit efflux pump activity was evaluated in *S. aureus* strains ATCC 25923, MRSA ATCC 43300, and MRSA 272123. This assessment was conducted using real-time fluorimetry by monitoring the intracellular accumulation of ethidium bromide, a known efflux pump substrate. Cell membrane damage effects of our compounds would also have resulted in increasing cellular concentrations of the dye. We observed no bacterial cell death under the assay conditions. All the used test concentrations did not even result in bacterial growth inhibition. So, effects on the cell membrane could be excluded from the results. Moreover, such effects would not have been dependent on structural changes within the compounds. Our observed effects showed a substituent dependency and were specific, as discussed.

The experiment was performed with an automated approach utilising a CLARIOstar Plus plate reader (BMG Labtech, Ortenberg, Germany). Positive controls of reserpine and carbonyl cyanide 3-chlorophenyl hydrazone (CCCP) were included at a concentration of 25 µM and 50 µM, respectively. The solvent DMSO was used at a subinhibitory concentration of 1% (*v*/*v*).

The bacterial cultures were grown in tryptic soy broth (TSB) until reaching an optical density (OD) of 0.4–0.6 at 600 nm. The cultures were then centrifuged at 13,000× *g* for 3 min, followed by washing and resuspension in PBS (pH 7.4). This centrifugation and resuspension process was repeated to ensure proper preparation of the bacterial suspension.

The tested compounds were applied at a concentration of 100 µM and were added to a 2 µg/mL ethidium bromide solution in PBS, a concentration that was nontoxic to the bacterial strains used. A total of 50 µL of this solution was dispensed into a black 96-well microtiter plate (Greiner Bio-One Hungary Kft, Mosonmagyaróvár, Hungary), followed by the addition of 50 µL of bacterial suspension (OD600 0.4–0.6) into each well.

Fluorescence measurements were recorded in real time using the CLARIOstar plate reader, with excitation and emission wavelengths set at 530 nm and 600 nm, respectively. Data collection was performed at one-minute intervals over a period of one hour. The inhibitory effect of the tested compounds was determined based on the relative fluorescence increase (*RFI*) at the 60 min mark, using the formula *RFI* = *RF*_treated_ − *RF*_untreated_/*RF*_untreated_, where *RF*_treated_ is the relative fluorescence (*RF*) at the last time point of the ethidium bromide accumulation curve in the presence of the compound, and *RF*_untreated_ is the *RF* at the last time point of the ethidium bromide accumulation curve of the untreated control (the well containing only DMSO).

However, in order to prove the concentration-dependent effect of the determined *RIF* values, most active inhibitors of each strain were measured at various concentrations. The results are shown in the Supplementary Materials.

**Assay for Enhancing the Antibacterial Growth Activity**

The minimum inhibitory concentrations (MICs) of the test compounds and antibiotics tetracycline and ciprofloxacin were determined using a microdilution technique according to the recommendations of the Clinical and Laboratory Standard Institute guidelines in *S. aureus* strains ATCC 25923, MRSA ATCC 43300 and MRSA 272123. MICs were determined in 96-well microtiter plates using a final volume of 100 µL Mueller Hinton Broth (MHB) containing beef infusion solids (20 g/L), casein hydrolysate (17.5 g/L) and starch (1.5 g/L). The antibiotics were dissolved in dimethyl sulfoxide (DMSO) and diluted with MHB to final concentrations of 100 µg/mL, containing 12.5% DMSO, except tetracycline in the *S. aureus* strains ATCC 25923 and MRSA ATCCC 43300, with a starting concentration of 1 µg/mL. DMSO should prevent precipitation during the following dilution procedures of the compounds, which easily dissolved in the solution. Further dilutions of the compounds and the antibiotics in the test medium were prepared at the required concentrations of 50, 25, 12.5, 6.25, 3.125, 1.56, 0.78, 0.39, 0.195, 0.0975 and 0.049 µg/mL, with MHB serially decreasing the final DMSO percentage, and of 0.5, 0.25, 0.125, 0.0625 and 0.03125 µg/mL, respectively. All the compounds and antibiotics were tested in triplicate for their in vitro growth inhibitory activity. In the antibacterial drug-enhancing studies, the compounds were used at 100 µM concentrations. The final inoculum size was 5 × 10^5^ CFU/mL for the antibacterial assay. A set of tubes containing only inoculated broth was used as a negative control. After incubation for 18 h at 37 ± 1 °C, the last tube with no growth of microorganisms determined through visual examination under an inverse microscope was recorded as the MIC (expressed in µg/mL).

**Assay for Cytotoxicity in Mammalian HEK293 Cells**

The human embryonic kidney cell line HEK293 (DSMZ Braunschweig, ACC305) was cultivated at 37 °C in a humidified incubator with 5% CO_2_ in Dulbecco’s Modified Eagle Medium (DMEM), with the following supplements: 10% FCS and 5 mM glutamine. The cells were seeded at a density of 1.5 × 10^3^ cells per well in a 96-well cell culture plate (TPP, Trasadingen, Switzerland). The compounds were added directly to the medium at increasing concentrations to determine the half-maximal inhibitory concentration (IC_50_) values. Following a 24 h incubation period, AlamarBlue^®^ reagent (Invitrogen, Carlsbad, CA, USA) was added in accordance with the manufacturer’s instructions and subsequently incubated for 4 h. Thereafter, the samples were subjected to analysis. The detection of viable cells, which convert resazurin (AlamarBlue reagent) into the highly fluorescent resorufin, was performed by utilising a FLUOstarOPTIMA microplate reader (BMG Labtec) with the following filter set: Ex 560 nm/Em 590 nm. All measurements were performed in triplicate.

## 4. Conclusions

There is a strong need for novel antibacterial compounds to combat the resistance against antibiotics that has partially developed to multidrug resistance (MDR), as reported for infections with *S. aureus*. Although the reasons for such drug resistances vary, the main reason in cases such as MDR is the expression of efflux pumps that lower bacterial drug levels and thus result in a resistance. In *S. aureus*, efflux pumps of almost all efflux pump families are known to transport all those antibiotics. Due to that, the finding of specific inhibitors of efflux pump activity will not be helpful. We succeeded in profiling a novel class of compounds of the benzo-anellated 1,4-dihydropyridine type as inhibitors of *S. aureus* efflux pumps, evaluated in an assay with a non-specific transporter substrate of efflux pump activity to cover most efflux pumps of *S. aureus*, including MRSA strains. We discovered the best activities against *S. aureus* in the group of those dibenzo-anellated compounds that have preferred 4-substituents at the 4-phenyl ring of the 1,4-dihydropyridine core, whereas those with additional pyrido residue had the best activities against all MRSA strains and the most favourable ones against the *S. aureus* strain. The determined nontoxicity of the most active compounds proved that they are specific compared to the known inhibitors reserpine and CCCP, which could only be used at the lower concentrations of 25 and 50 µM, respectively, due to toxic problems.

Finally, the best compound activities of efflux pump inhibitors were verified in *S. aureus* strains to increase the determined antibiotic activities and reduce MIC values for tetracycline and ciprofloxacin as a proof of concept that the inhibition of efflux pumps really increased the effectiveness of the used antibiotics. So, our novel compound class is a novel class of compounds with respective activities towards various *S. aureus* strains, thus contributing to combatting the MDR phenomenon.

## Figures and Tables

**Table 1:** Efflux pump-inhibiting data as *RFI* values, with SD values in brackets, for target compound series **4a**–**g**, **5a**–**c**, **9**, **10a**–**d** and **13a**–**c**, with varied substitution patterns in *S. aureus* ATCC 25923 and MRSA strains.

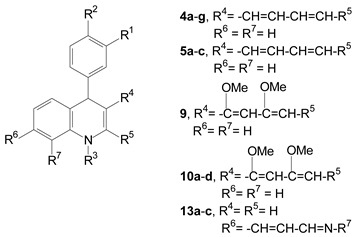						
				*RFI* ^a^		
Comp.	R^1^	R^2^	R^3^	*S. aureus*	MRSA 43300	MRSA 272123
**4a**	Ome	H	Bn-3-COOMe	0.1 (0.08)	0.04 (0.01)	0.86 ^e^ (0.01)
**4b**	H	OMe	Bn-3-COOMe	0.6 (0.32)	0.03 (0.01)	0.12 (0.07)
**4c**	OMe	OMe	Bn-3-COOMe	0.02 (0.04)	0.06 (0.02)	0.14 (0.08)
**4d**	OMe	OMe	Bn-4-COOMe	0.12 (0.07)	n.a. ^b^	0.19 (0.08)
**4e**	Cl	H	Bn-3-COOMe	n.a. ^b^	0.04 (0.01)	0.08 (0.01)
**4f**	H	Cl	Bn-3-COOMe	n.a. ^b^	0.01 (0.005)	0.16 (0.05)
**4g**	Cl	Cl	Bn-3-COOMe	1.57 ^e^ (0.46)	n.a. ^b^	0.05 (0.01)
**5a**	Cl	H	Me	n.a. ^b^	0.11 ^d^ (0.01)	0.30 ^d^ (0.1)
**5b**	H	Cl	Me	0.03 (0.01)	0.08 (0.04)	0.18 (0.1)
**5c**	Cl	Cl	Me	0.09 (0.03)	0.10 (0.05)	0.14 (0.07)
**9**	H	Cl	Bn	n.a. ^b^	0.03 (0.01)	0.20 ^d^ (0.1)
**10a**	Cl	H	Me	n.a. ^b^	0.11 (0.01)	0.23 ^d^ (0.06)
**10b**	H	Cl	Me	0.01 (0.005)	0.12 (0.07)	0.11 (0.06)
**10c**	Cl	Cl	Me	0.11 (0.05)	0.09 (0.03)	0.16 (0.08)
**10d**	OMe	OMe	Me	0.01 (0.006)	0.01 (0.006)	0.09 (0.01)
**13a**	H	Cl	Bn	0.19 ^d,e^ (0.07)	0.21 ^e^ (0.02)	0.24 ^d^ (0.1)
**13b**	H	Cl	Bn-4-OMe	0.21 ^d,e^ (0.06)	0.28 ^d,e^ (0.06)	0.28 ^d^ (0.04)
**13c**	Cl	Cl	Bn-4-OMe	0.16 ^d,e^ (0.05)	0.26 ^d,e^ (0.03)	0.36 ^d^ (0.1)
reserpine				0.82 (0.2)	0.14 (0.06)	0.28 (0.07)
CCCP				0.73 (0.3)	0.22 (0.09)	n.d. ^c^

^a^ Mean of three determinations. ^b^ Not active. ^c^ Not determined. ^d^ Statistically significant compared to the DMSO control at *p* < 0.05 after Welch *t*-test and Holm correction. ^e^ Statistically significant compared to the DMSO control at *p* < 0.05 after ANOVA test with Dunnett post hoc test.

**Table 2:** MIC values for tetracycline and ciprofloxacin alone and with efflux pump inhibitors **4b**, **4g**, **5a**, **13b** and **13c** in *S. aureus* ATCC 25923 and MRSA strains, with factors of reduction in brackets.

	MIC [µg/mL] ^a^		
Comp.	*S. aureus*	MRSA 43300	MRSA 272123
tetracycline	0.0625	0.031	25
tetracycline + **4b**	<0.0019 (>32)	n.d. ^b^	12.5 (2)
tetracycline + **4g**	<0.0019 (>32)	n.d. ^b^	25
tetracycline + **5a**	n.d. ^b^	0.031	12.5 (2)
tetracycline + **13b**	<0.0019 (>32)	<0.0019 (16)	25
tetracycline + **13c**	<0.0019 (>32)	0.031	25
ciprofloxacin	0.78	0.39	25
ciprofloxacin + **4b**	<0.097 (>8)	n.d. ^b^	12.5 (2)
ciprofloxacin + **4g**	<0.097 (>8)	n.d. ^b^	25
ciprofloxacin + **5a**	n.d. ^b^	0.39	12.5 (2)
ciprofloxacin + **13b**	<0.097 (>8)	<0.097 (>4)	25
ciprofloxacin + **13c**	0.39 (2)	<0.097 (>4)	25

^a^ Mean of three determinations. ^b^ Not determined.

## Data Availability

No new data were created or analyzed in this study. Data sharing is not applicable to this article.

## Supplementary Materials

The following supporting information can be downloaded at: https://www.mdpi.com/article/10.3390/ijms27093738/s1.
