# Supplementary material for: Evaluation of Novel Benzo-Annelated 1,4-Dihydropyridines as Potential Inhibitors of Antibacterial Efflux Pumps in S. aureus and MRSA Strains
Source: Int J Mol Sci. 2026 Apr 23;27(9):3738. doi: 10.3390/ijms27093738 (PMC13164449; doi:10.3390/ijms27093738)
Supplement: Supplementary file 1 [file ijms-27-03738-s001.zip › ijms-4212963-supplementary.pdf]

Supplementary Information

Communication

Evaluation of Novel Benzo-Annelated 1,4-Dihydropyridines as Potential Inhibitors of Antibacterial Efflux Pumps in *S. aureus* and MRSA strains

Peter Werner [1], Nikoletta Szemerédi [2], Gabriella Spengler [2], Frank Erdmann [1] and Andreas Hilgeroth [1,] *

Table S1. Concentration-dependent effects of the *RIF* values for the most active compounds determined in the used *S. aureus* strains.

	RIF value						
	S. aureus		MRSA 43300			MRSA 272123	
Concentration [µM]	**4b**	**13b**	**4b**	**13b**	**13c**	**4b**	**13c**
10	0.25	0.05	0.07	0.08	0.04	0.13	0.01
25	0.36	0.03	0.2	0.19	0.13	0.16	0.11
50	0.92	0.06	0.04	0.1	0.14	0.12	0.13
150	1.01	0.06	0.51	0.14	0.24	0.10	0.16
200	1.10	1.75	1.16	0.26	0.35	0.19	0.14
